# Osteogenic Differentiation of Three-Dimensional Bioprinted Constructs Consisting of Human Adipose-Derived Stem Cells *In Vitro* and *In Vivo*

**DOI:** 10.1371/journal.pone.0157214

**Published:** 2016-06-22

**Authors:** Xiao-Fei Wang, Yang Song, Yun-Song Liu, Yu-chun Sun, Yu-guang Wang, Yong Wang, Pei-Jun Lyu

**Affiliations:** Center of Digital Dentistry, Faculty of Prosthodontics, Peking University School and Hospital of Stomatology & National Engineering Laboratory for Digital and Material Technology of Stomatology & Research Center of Engineering and Technology for Digital Dentistry of Ministry of Health, Beijing 100081, China; University of Sheffield, UNITED KINGDOM

## Abstract

Here, we aimed to investigate osteogenic differentiation of human adipose-derived stem cells (hASCs) in three-dimensional (3D) bioprinted tissue constructs *in vitro* and *in vivo*. A 3D Bio-plotter dispensing system was used for building 3D constructs. Cell viability was determined using live/dead cell staining. After 7 and 14 days of culture, real-time quantitative polymerase chain reaction (PCR) was performed to analyze the expression of osteogenesis-related genes (*RUNX2*, *OSX*, and *OCN*). Western blotting for *RUNX2* and immunofluorescent staining for *OCN* and *RUNX2* were also performed. At 8 weeks after surgery, osteoids secreted by osteogenically differentiated cells were assessed by hematoxylin-eosin (H&E) staining, Masson trichrome staining, and *OCN* immunohistochemical staining. Results from live/dead cell staining showed that most of the cells remained alive, with a cell viability of 89%, on day 1 after printing. *In vitro* osteogenic induction of the 3D construct showed that the expression levels of *RUNX2*, *OSX*, and *OCN* were significantly increased on days 7 and 14 after printing in cells cultured in osteogenic medium (OM) compared with that in normal proliferation medium (PM). Fluorescence microscopy and western blotting showed that the expression of osteogenesis-related proteins was significantly higher in cells cultured in OM than in cells cultured in PM. *In vivo* studies demonstrated obvious bone matrix formation in the 3D bioprinted constructs. These results indicated that 3D bioprinted constructs consisting of hASCs had the ability to promote mineralized matrix formation and that hASCs could be used in 3D bioprinted constructs for the repair of large bone tissue defects.

## Introduction

Dental caries, periodontal disease, dental trauma, cancer, and other diseases can lead to maxillofacial bone defects, which are commonly encountered by dentists [1]. Currently, tissue engineering has been frequently applied for the treatment of bone defects. Tissue engineering involves three necessary elements [2–4]: cells with high osteogenic potential; osteogenic growth factors, and a 3D scaffold that is porous for vascularization and gives the 3D construct sufficient mechanical properties for loading. To form an ideal construct, the seeded cells should be autologous and easy to obtain; the scaffold should be biodegradable and derived from homologous materials [5].

Stem cells derived from bone marrow (BMSCs) and adipose tissue (ASCs) possess the capabilities of self-renewal and differentiation into osteoblasts. However, the minimal invasive capacity, ease of access, and abundance of hASCs in adipose tissue provide clear advantages over BMSCs and make these stem cells an ideal source for tissue engineering therapies [6]. Therefore, human ASCs (hASCs) may have applications in tissue engineering as seed cells.

In addition, a biofabrication approach that is able to generate a 3D blueprint of the patient’s specific disorder is needed in order to restore the functionality of the tissue and repair the defect using autologous cells. Traditional tissue engineering techniques involve seeding cells onto a scaffold to form a cell-scaffold complex, followed by *in vitro* cultivation or *in vivo* implantation into the corresponding lesion. Precise control over the cellular distribution and density within the scaffold is difficult to achieve. 3D bioprinting is a new tissue engineering method that applies rapid prototyping (RP) techniques [7–9]. These RP techniques follow computer-assisted design to build a complex 3D tissue construct. Through this method, 3D bioprinting has great potential to fabricate tissues with multiple biocomposite materials and cell types, all of which are extremely important for the advancement of bone tissue engineering. Moreover, 3D porous scaffolds are more conducive to cell and matrix interactions. Compared with nonporous materials, the 3D porous scaffolds owned lots of pores, which could transport oxygen and nutrients for cells, and moreover, these pores can promote the growth of blood vessels into the scaffold materials [10,11]. Furthermore, enhanced oxygen, nutrient, and waste diffusion are plausible [12,13]. Although many studies have examined osteogenic differentiation within bioprinted tissue constructs [14–16], few studies have previously examined the osteogenic differentiation of 3D constructs consisting of hASCs *in vitro* and *in vivo*.

Therefore, in this study, we aimed to establish a framework for the development of bioprinting in the field of bone regeneration using hASCs for bone tissue engineering combined with 3D bioprinting techniques. We investigated the *in vitro* and *in vivo* osteogenic differentiation of hASC bioprinted tissue constructs. This technique is expected to provide a reference for regenerative therapy of maxillofacial and systemic bone defects.

## Materials and Methods

### Ethical considerations

Animal welfare and experimental procedures were carried out in accordance with the Guide for the Care and Use of Laboratory Animals (Ministry of Science and Technology of China, 2006) and were approved by the animal ethics committee of Peking University, China (LA2014227).

### Materials

hASCs were purchased from ScienCell Research Laboratories (USA). Low viscosity alginate, gelatin, Alizarin Red S, Triton X-100, sodium dodecyl sulfate (SDS), calcium chloride, phalloidin, osteogenic differentiation inducing factor β-glycerophosphate disodium salt hydrate, ascorbic acid, and dexamethasone were purchased from Sigma-Aldrich (USA). Dulbecco’s modified Eagle’s medium (DMEM), fetal bovine serum (FBS), and 100× penicillin-streptomycin mixture for cell culture were purchased from Gibco (USA). Cell culture dishes and other consumables were purchased from Corning Incorporated (USA). Calcein-AM and propidium iodide were purchased from DOJINDO (Japan).

### Cell culture and cell differentiation assays

At passage 3 (P3), hASCs were cultured in PM containing DMEM with 100 U/mL penicillin G, 100 mg/mL streptomycin, and 10% FBS at 37°C in an incubator with an atmosphere comprising 95% air, 5% CO_2_, and 100% relative humidity [17]. All cell-based experiments were repeated at least twice.

Cells were seeded in osteogenic medium (OM) containing 10 mM β-glycerophosphate, 100 nM dexamethasone, and 200 mM ascorbic acid to induce osteogenesis. The level of alkaline phosphatase (ALP) activity was examined on day 7 using an ALP kit according to the manufacturer’s protocol. Mineralization was determined by staining with Alizarin red S on day 14.

### Preparation of the hydrogel

A hydrogel consisting of gelatin (cat. no. 53028; Sigma) and alginate (cat. no. A0682; Sigma) was used. The viscosity of alginate was 4–12 CP. We used 8 wt% alginate and 2 wt% gelatin dissolved in NaCl solution under constant stirring at 40°C. These concentrations of alginate and gelatin gave a suitable viscosity for 3D bioprinting. This yielded 10 wt% hydrogel [18,19]. The prepared solution was sterilized by incubation at 70°C for 30 min three times. hASCs were dissociated to single cells, suspended in proliferation medium, and then gently mixed with the hydrogel to reach a final concentration of 3 × 10^6^ cells/mL [20].

### Evaluation of hydrogel porosity

The hydrogel were dehydrated with a graded series of ethanol, dried in a critical point dryer (Micro Modul YO-230; Thermo Scientific, Waltham, MA, USA), mounted onto aluminum stubs, sputter coated with gold, and viewed under a field emission scanning electron microscope (FE-SEM; Hitachi S4800; Hitachi, Japan) for observation of the pore size of the hydrogel.

To determine the microporosity of the hydrogel, a liquid displacement method was used according to previously reported methods [21]. Five replicates of the hydrogel were used, and the data represent the average values obtained.

### 3D bioprinting

**Fig 1:**
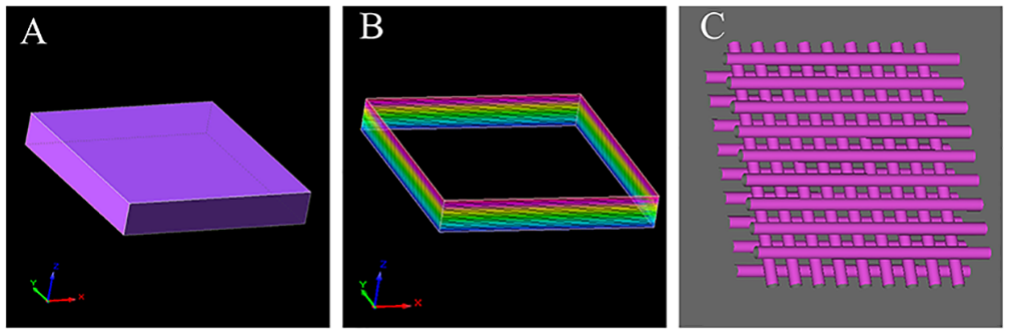
Design of the 3D printed tissue construct. A: Regular geometry of the printed tissue construct. B: Blueprint recognizable by 3D Bio-plotter. C: Diagram of the inner grid structure of 3D printed tissue construct: 0–90°.

A 3D Bio-plotter dispensing system (Envision Tec, Germany) was used for building 3D constructs [22]. A cuboid model was designed and converted into a vector file format, as shown in Fig 1A and 1B. The distance between each printed line was 1.5 mm. The inner structure was 0–90°, as shown in Fig 1C. Alginate/gelatin scaffolds were fabricated using layer-by-layer deposition. When each print was completed, the final constructs were ionically cross-linked in CaCl_2_ (200 mM) for 5 min. The parameters of the 3D Bio-plotter were determined after 37 repeated experimentations in order to ensure the consistency and repeatability of the 3D bioprinted constructs. Related parameters are presented in Table 1.

**Table 1:** Key parameters of the 3D Bio-plotter.

Parameter	Temperature (°C)	Needle description (mm)	Pressure (bar)	Speed (mm/s)	Platform temperature (°C)
**Reference value**	35–37	0.25	2.0–3.0	10.0	4

The printed tissue constructs were placed in a 6-cm dish containing 2 mL OM or 2 mL PM. 3D constructs were cultured for 1 day, 7 days and 14 days respectively before evaluation. Medium was changed three times a week.

### Observation of the cell distribution in the 3D bioprinted tissue construct

An inverted light microscope was used to observe the 3D bioprinted tissue construct and cellular morphology in the hydrogel. Phalloidin was applied to perform immunofluorescent staining of cytoskeletal proteins within the 3D printed tissue construct according to the manufacturer’s protocol. Next, cells were stained with DAPI for nuclear staining and visualized using a confocal laser-scanning microscope (CLSM; TLS SP2; Leica, Wetzlar, Germany). 3D reconstruction software was used to reconstruct the 3D spatial distribution of the cells within the tissue construct.

### Cell viability assay in 3D constructs

Cell viability in the 3D bioprinted constructs was determined on days 1 and 7 after printing using Calcein-AM (CAM) and propidium iodide (PI). The printed constructs were incubated in proliferation medium with a concentration of 5 μmol/L CAM and 3 μmol/L PI for 45 min at 37°C. The constructs were then washed with PBS three times, and stained cells (green fluorescence for live cells, red fluorescence for dead cells) were visualized using a CLSM (TLS SP2; Leica). Live and dead cells were counted using Image Pro Plus 6.0 software, and cell viability and live cell numbers on days 1 and 7 were determined as follows:

Cell viability=100%×livecells/(live cells+dead cells)

### Cell adhesion in tissue constructs

Cell vinculin was detected on days 1 and 7 after printing to observe cellular adherence and growth. The printed 3D constructs were washed three times with phosphate-buffered saline (PBS), and immunofluorescent staining for vinculin was performed according to the manufacturer’s protocol (Cell Signaling Technology, Danvers, MA, USA). After staining for vinculin, the cells were counterstained with DAPI for nuclear staining and visualized using a CLSM (TLS SP2; Leica).

### Real-time quantitative reverse transcription polymerase chain reaction (qRT-PCR)

The printed 3D constructs cultured for 7 or 14 days were analyzed for gene expression (n = 3). Total RNA was extracted and reverse transcribed according to the manufacturer’s instructions (Invitrogen, Carlsbad, CA, USA). Real-time quantitative PCR was performed according to the manufacturer’s protocol (KAPA Biosystems, USA). The primers for Runt-related transcription factor 2 (*RUNX2*), osterix (*OSX*), and osteocalcin (*OCN*) were synthesized by Invitrogen (Table 2). β-Actin was used as an internal standard. The cycle threshold values (Ct values) were used to calculate the fold differences by the ΔΔCt method.

**Table 2:** Sequences of the primers used for real-time PCR.

Genes	Forward primer	Reverse primer
*RUNX2*	CGCATTCCTCATCCCAGTAT	AGGGGTAAGACTGGTCATAGGA
*OCN*	CTGTATCAATGGCTGGGAGC	GCCTGGAGAGGAGCAGAACT
*OSX*	GTGCAAGGCACTATGCTAGATC	CGTTACAGGAAAGGCACGAA
*β-Actin*	AGCACAATGAAGATCAAGATCAT	ACTCGTCATACTCCTGCTTGC

### Immunofluorescent staining for *OCN* and *RUNX2*

After 14 days of culture, the printed 3D constructs of the two groups were rinsed three times with PBS, and immunofluorescent staining for *OCN* and *RUNX2* was then performed using specific antibodies (Cell Signaling Technology), according to the manufacturer’s protocol. After staining for *OCN* and *RUNX2*, the cells were counterstained with DAPI for nuclear staining and visualized using a CLSM (TLS SP2; Leica).

### Western blotting for detection of *RUNX2* protein levels

After 14 days of culture, the total protein from cells within PM and OM printed constructs (n = 4) was extracted for protein quantification. Protein concentrations were determined for all extracts and adjusted accordingly. Electrophoresis was carried out with 5% stacking gels and 10% separating gels using 6 μg protein per well. Subsequently, wet transfer onto the membranes was performed, followed by blocking, and incubation with *RUNX2* primary antibodies (Cell Signaling Technology). After incubation, membranes were washed, incubated with secondary antibody, washed again, and subjected to development and imaging.

### Ectopic bone formation *in vivo*

Implants were divided into two groups: printed 3D constructs without hASCs and 3D constructs with hASCs. After 7 days of *in vitro* culture, 7-week-old male BALB/c nude mice were anaesthetized with pentobarbital, and the above implants were placed aseptically into the dorsal subcutaneous area. At 8 weeks after surgery, nude mice(n = 8 per group) were sacrificed by cervical dislocation,and the implants were harvested (n = 8 implants for each group). The implants were fixed with 4% paraform aldehyde, dehydrated, and embedded in paraffin. Paraffin-embedded sections were then stained with hematoxylin and eosin (H&E) and Masson trichrome staining. Osteogenesis was evaluated by immunohistochemistry (IHC) for *OCN*.

### Statistical analysis

Data were analyzed using SPSS 17.0. After confirmation of homogeneity of variance, one-way analysis of variance (ANOVA) plus the least significant difference (LSD) test were performed for overall evaluation of cell viability and relative quantification of RNA expression in each group. The significance level was α = 0.05, and all tests were two-tailed.

## Results

### hASC cultivation and evaluation of osteogenic differentiation ability

**Fig 2:**
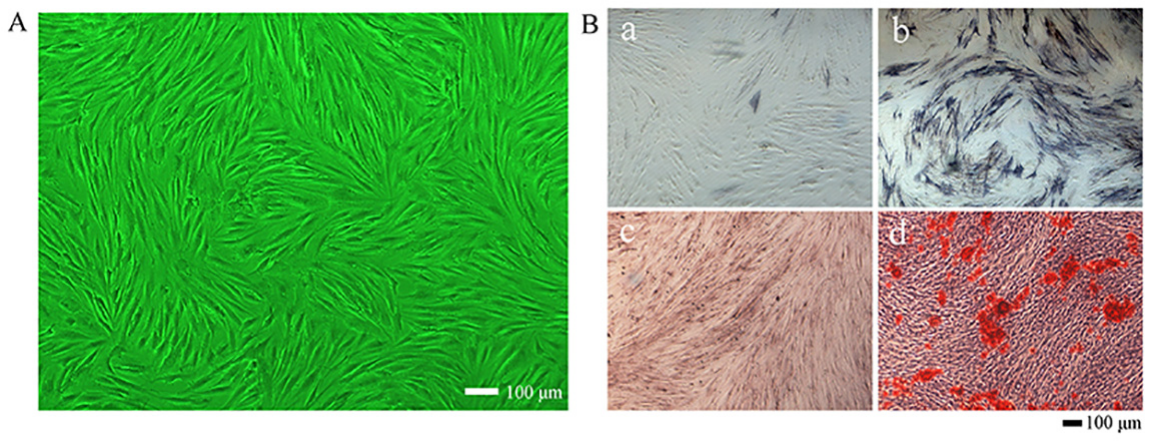
Evaluation of the osteogenic differentiation capacity of hASCs. A: hASCs at P3 showing fibroblast-like adherent growth. B (a): Negative ALP staining for cells cultured in PM. B (b): Positive ALP staining for cells cultured in OM. B (c): Negative Alizarin red S staining for cells cultured in PM. B (d): Positive Alizarin red S staining for cells cultured in OM. (40×).

At P3, hASCs showed fibroblast-like adherent growth (Fig 2A) with regular morphology. Cell counts of hASCs reached 4 × 10^6^ cells in all 10-cm cell culture dishes. After 7 days in OM, hASCs showed positive ALP staining (Fig 2Bb). After 14 days in OM, Alizarin red S staining revealed the formation of mineralization nodules (Fig 2Bd). The PM groups showed negative ALP staining results after 7 days and negative Alizarin red S staining results after 14 days (Fig 2Ba and 2Bc).

### Porosity of the hydrogel

**Fig 3:**
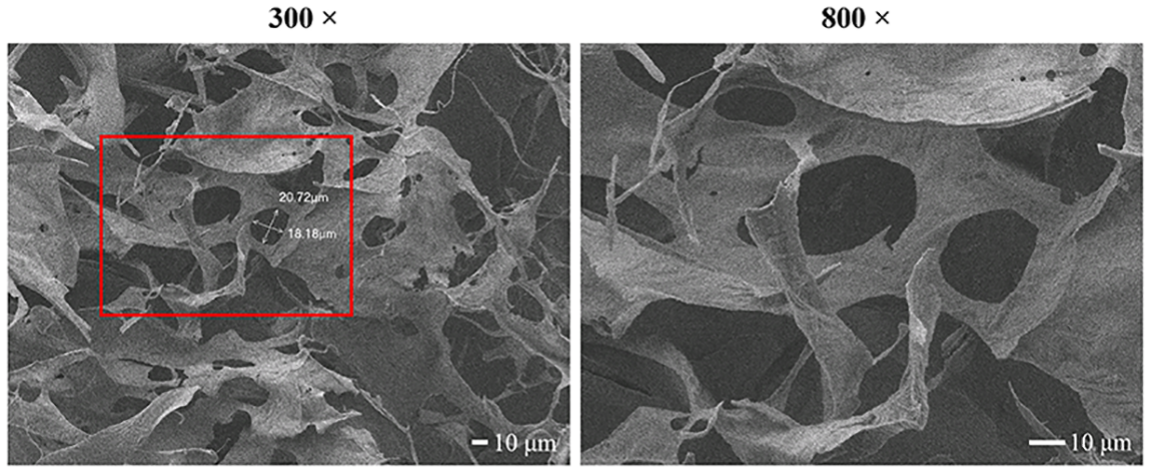
SEM images showing the porous internal structure of the alginate/gelatin hydrogel (see S1 Fig).

SEM images showed that the hydrogel consisting of alginate and gelatin had an uneven porous internal structure (Fig 3). The average diameter of the pores was 28.47 ± 3.56 μm. The porosity of the scaffolds was determined by a liquid displacement method using ethanol as the displacement fluid. The average porosity value of the hydrogel was 24.18% ± 7.9%.

### 3D distribution of hASCs within 3D bioprinted constructs

**Fig 4:**
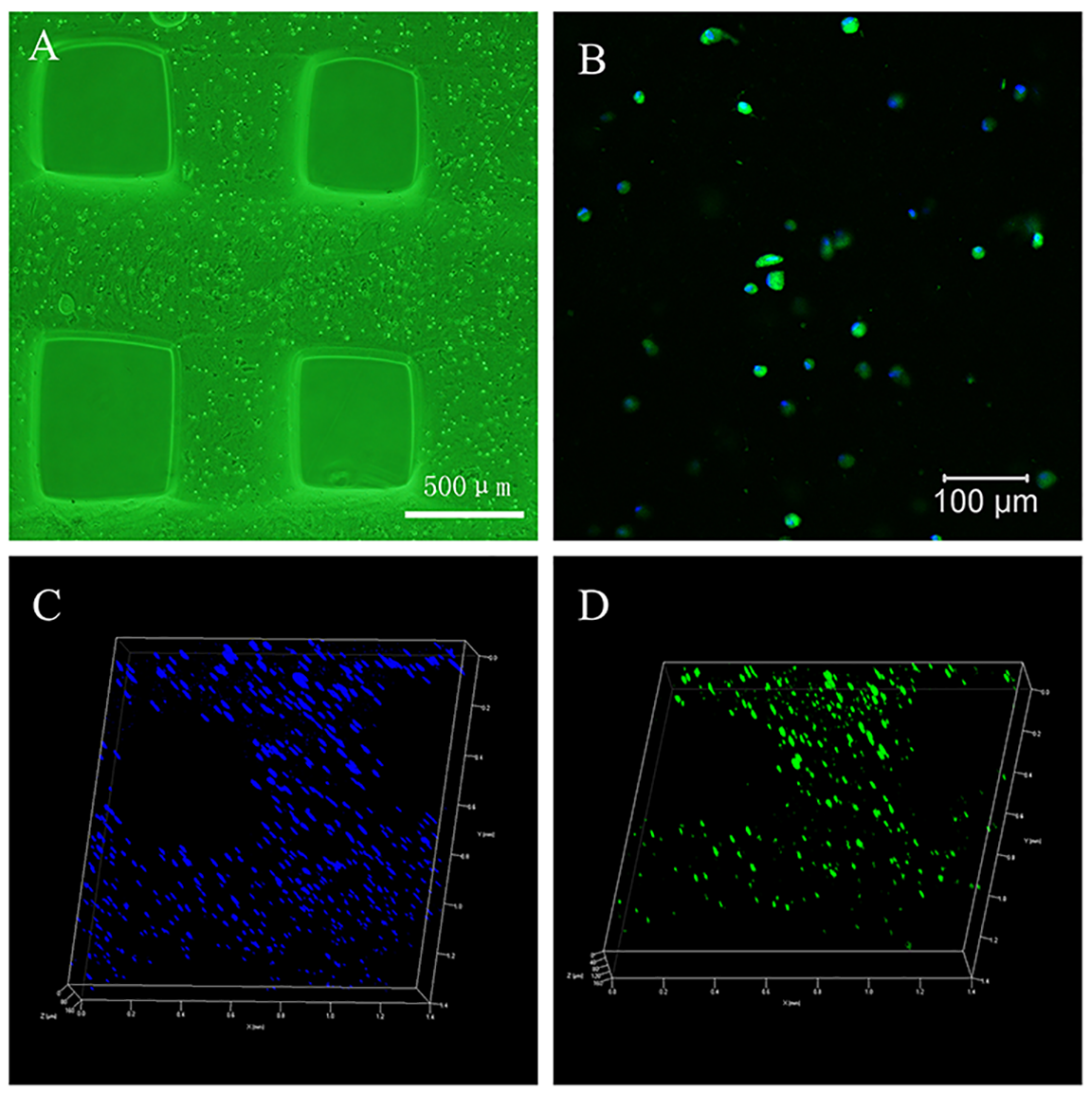
Distribution of hASCs in the bioprinted tissue constructs. A: Uniform cellular distribution in the construct (40×). B: Cells suspended in hydrogel (100×). C: DAPI-stained nuclei indicating cellular 3D spatial distribution (50×, 3D). D: Phalloidin-stained cytoskeletal proteins indicating cellular 3D spatial distribution (50×, 3D) (see S2 and S3 Figs).

The 3D bioprinted construct had a regular shape. hASCs were uniformly distributed within the 3D hydrogel scaffold (Fig 4A). The cells were elongated spindle-like cells and suspended within the hydrogel. The cellular morphology was consistent with observation of cells under a fluorescence microscope (Fig 4B). Confocal fluorescence microscopy revealed a uniform cellular distribution within the printed tissue construct, while cytoskeletal protein and nuclear staining revealed that a high density of viable cells was present within the 3D constructs (Fig 4C and 4D).

### Cell viability after bioprinting in 3D tissue constructs

**Fig 5:**
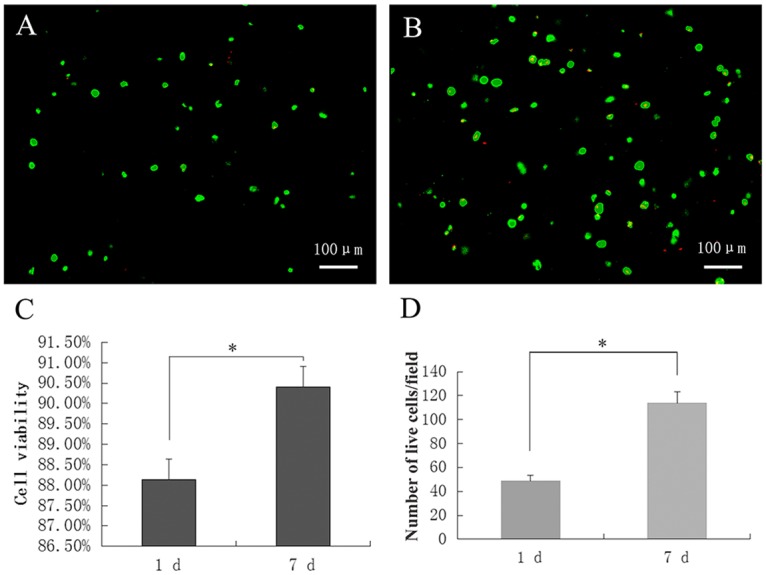
Immunofluorescent staining for live and dead cells in 3D constructs on days 1 and 7 after printing. Live cells were stained green, and dead cells were stained red. A: Day 1 after printing (100×). B: Day 7 after printing (100×). C: Cell viability in the constructs on days 1 and 7 after printing. D: Number of live cells in the construct on days 1 and 7 after printing. *P< 0.05.

CAM and PI immunofluorescent staining for live and dead cells showed that most cells were stained with green fluorescence, whereas the few dead cells present were stained with red fluorescence (Fig 5A and 5B). The live and dead cells were counted, and cell viability was calculated on days 1 and 7 after printing. The results showed that the cell viability was 88.13% on day 1 and 90.41% on day 7; this difference was statistically significant (*P*<0.05; Fig 5C). The number of viable cells on day 7 was greater than that on day 1 (*P*<0.05; Fig 5D).

### Growth of hASCs in the 3D bioprinted construct

**Fig 6:**
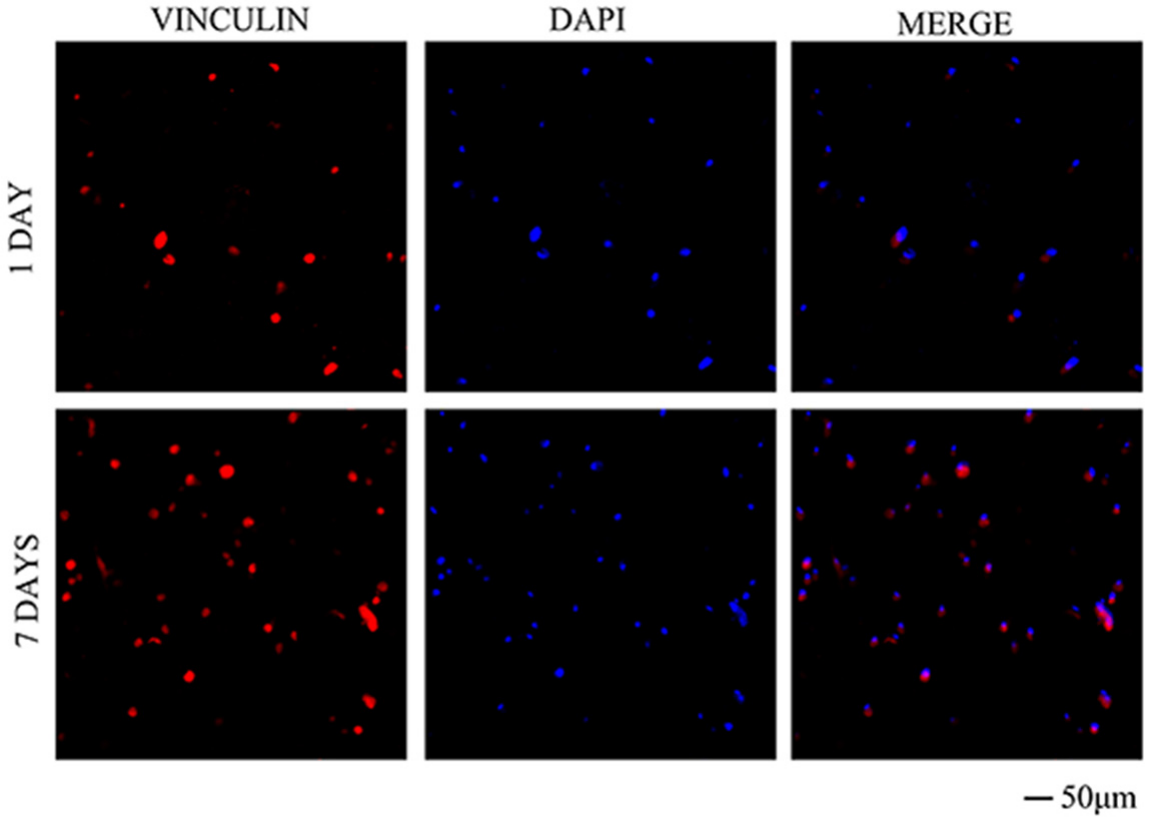
Immunofluorescent staining for vinculin in 3D constructs on days 1 and 7 after printing (200×).

**Fig 7:**
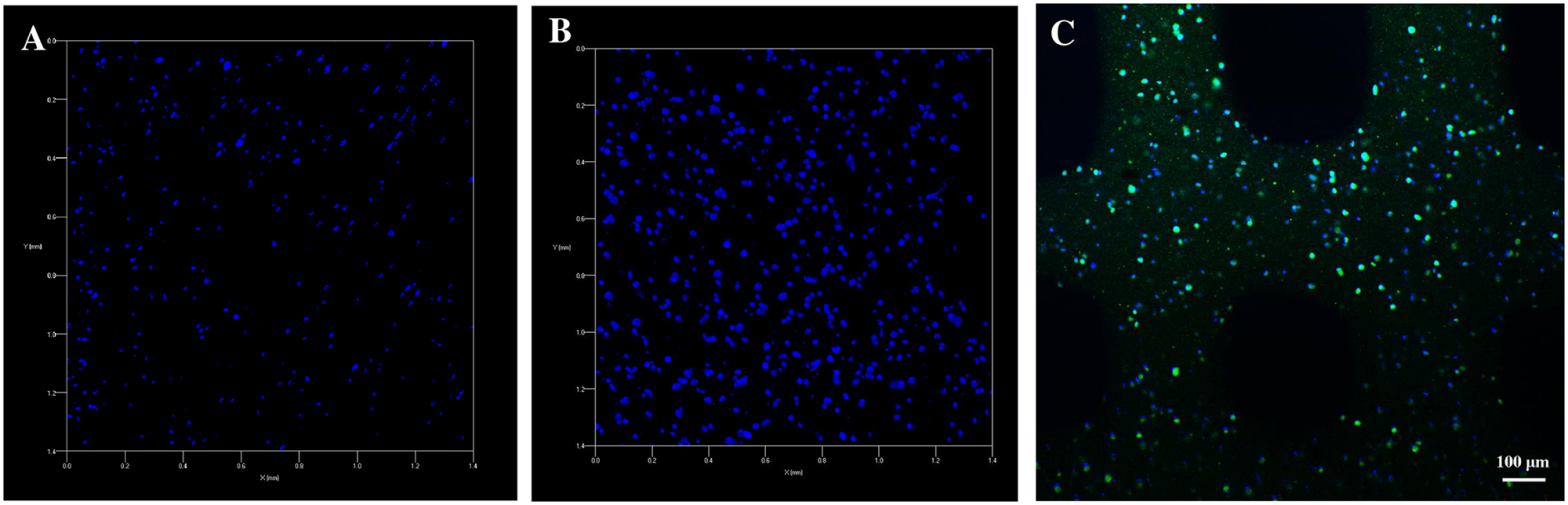
Cell proliferation in the printed tissue constructs. A: DAPI-stained nuclei indicating the cellular 3D spatial distribution on day 1 (100×, 3D). B: DAPI-stained nuclei indicating the cellular 3D spatial distribution on day 7 (100×, 3D). C: The 3D construct printed after 7 days (50×, 2D).

Immunofluorescent staining showed that vinculin protein was secreted by cells within the hydrogel on day 1 after printing, and the expression of vinculin was increased on day 7 after printing (Fig 6), indicating that cells could grow normally in the hydrogel scaffold. Results of immunofluorescent staining of DAPI on days 1 and 7 after printing were consistent with the results of live/dead cell staining. Direct visual observation showed that the cell count was higher on day 7 (Fig 7B) than on day 1 (Fig 7A) after printing. Moreover, the construct could still maintain the original structure, as shown in Fig 7C.

### Osteogenic differentiation of 3D bioprinted constructs *in vitro*

**Fig 8:**
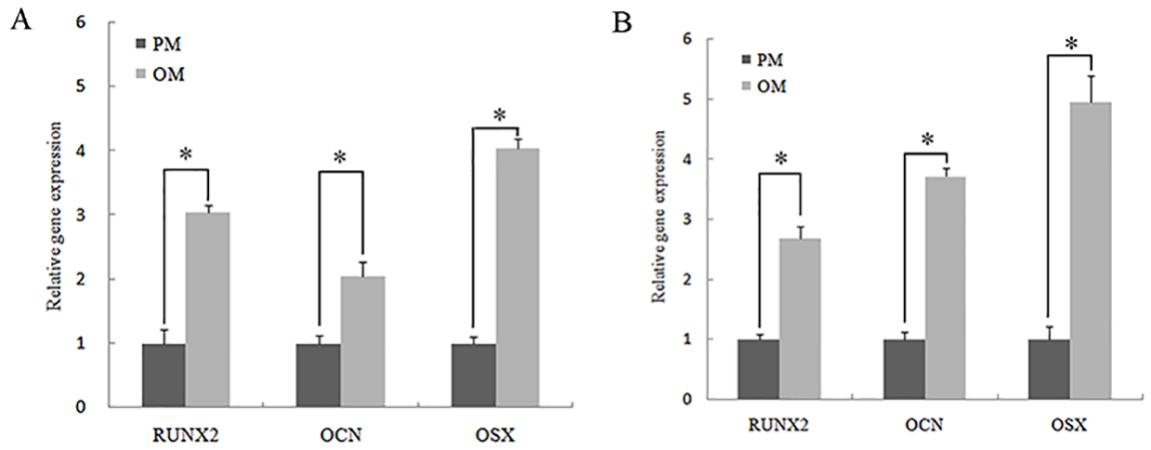
Expression of *RUNX2*, *OCN*, and *OSX* genes in hASCs cultured in PM or OM. A: 7 days after printing. B: 14 days after printing. *P< 0.05.

**Fig 9:**
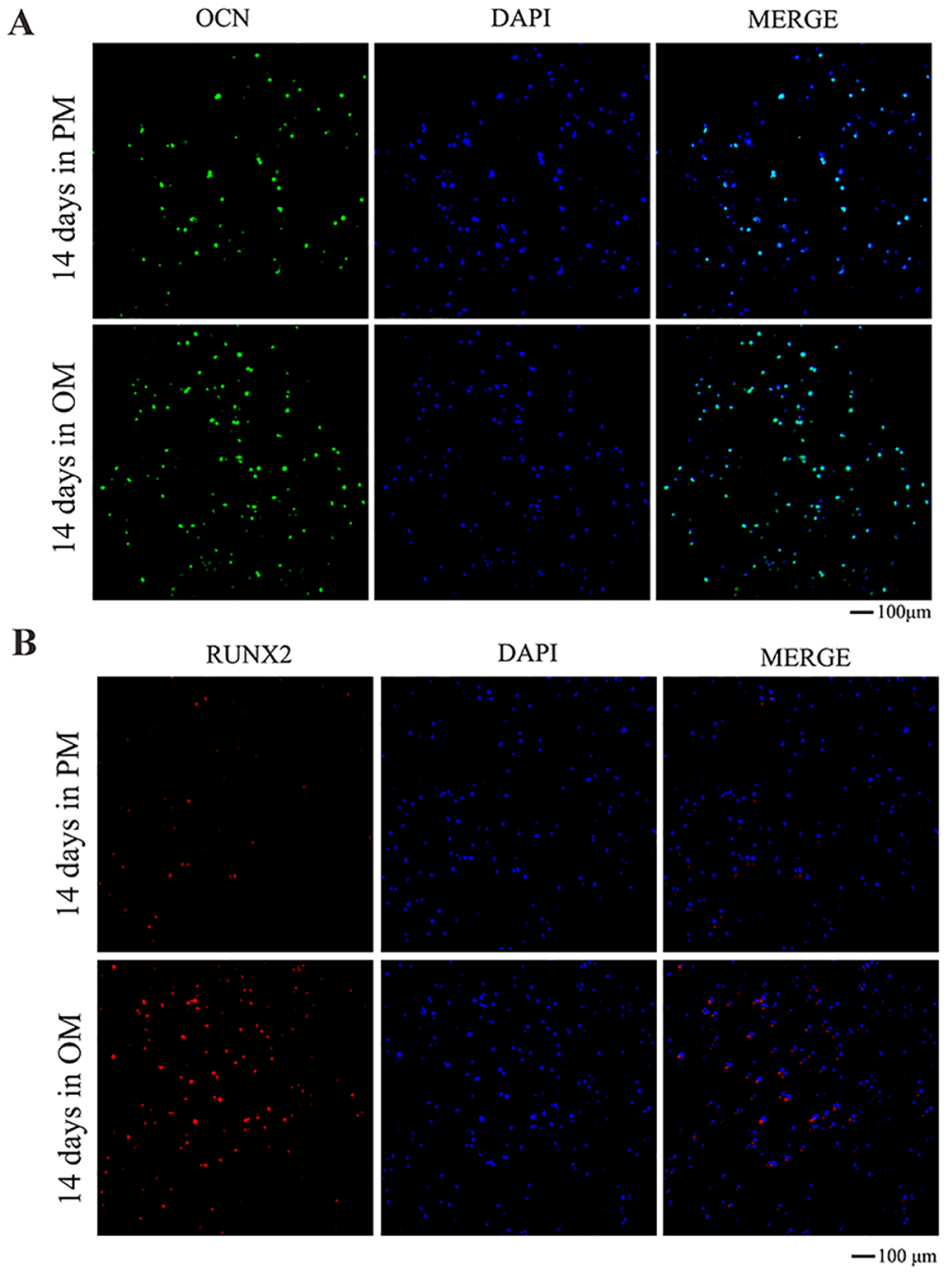
Immunofluorescent staining for osteogenic proteins in hASCs 3D constructs cultured in PM or OM for 14 days. A: Immunofluorescent staining for *OCN* (100×). B: Immunofluorescent staining for *RUNX2* (100×).

**Fig 10:**
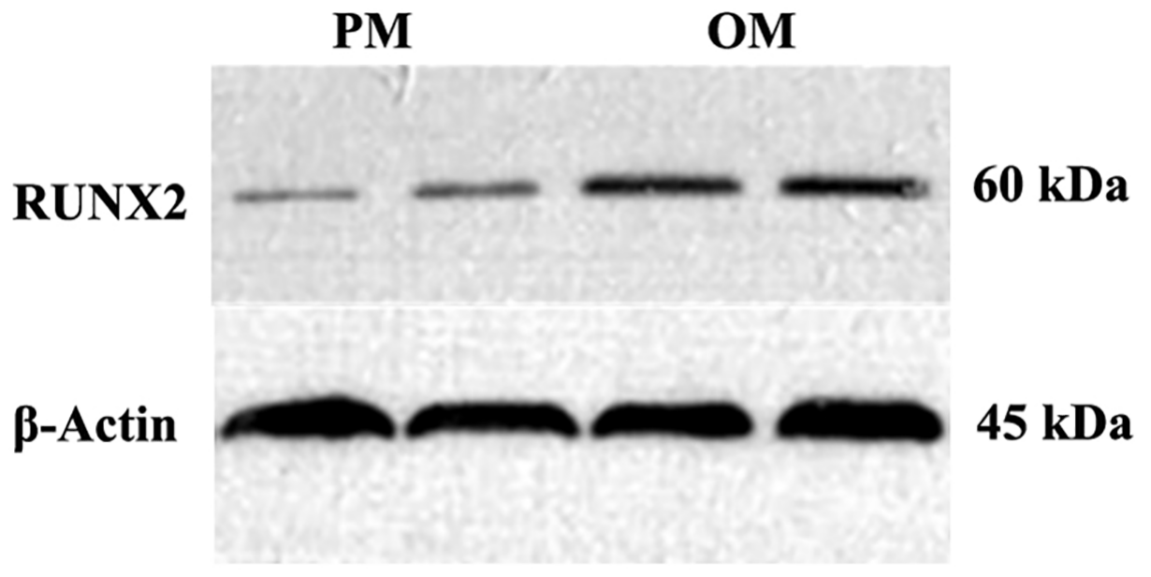
Western blotting for detection of *RUNX2* in the 3D constructs cultured in PM or OM for 14 days.

The results of qRT-PCR revealed that after being cultured in OM, the expression levels of osteogenic genes (*OSX*, *RUNX2*, and *OCN*) were significantly increased (*P*< 0.05) compared with those in constructs cultured in PM both on days 7 (Fig 8A) and 14 after printing (Fig 8B). Immunofluorescent staining for *OCN* showed that constructs cultured in OM produced more *OCN* protein after 14 days than constructs cultured in PM; however, DAPI staining revealed that there were no significant differences between constructs cultured in OM and PM (Fig 9A). The results of immunofluorescent staining for *RUNX2* on day 14 after printing were consistent with the results of immunofluorescent staining for *OCN* (Fig 9B). Moreover, western blotting showed that the level of *RUNX2* protein secretion was significantly increased in constructs cultured in OM (*P*< 0.05) compared with that in constructs cultured in PM (Fig 10).

### Ectopic bone formation in 3D bioprinted constructs *in vivo*

**Fig 11:**
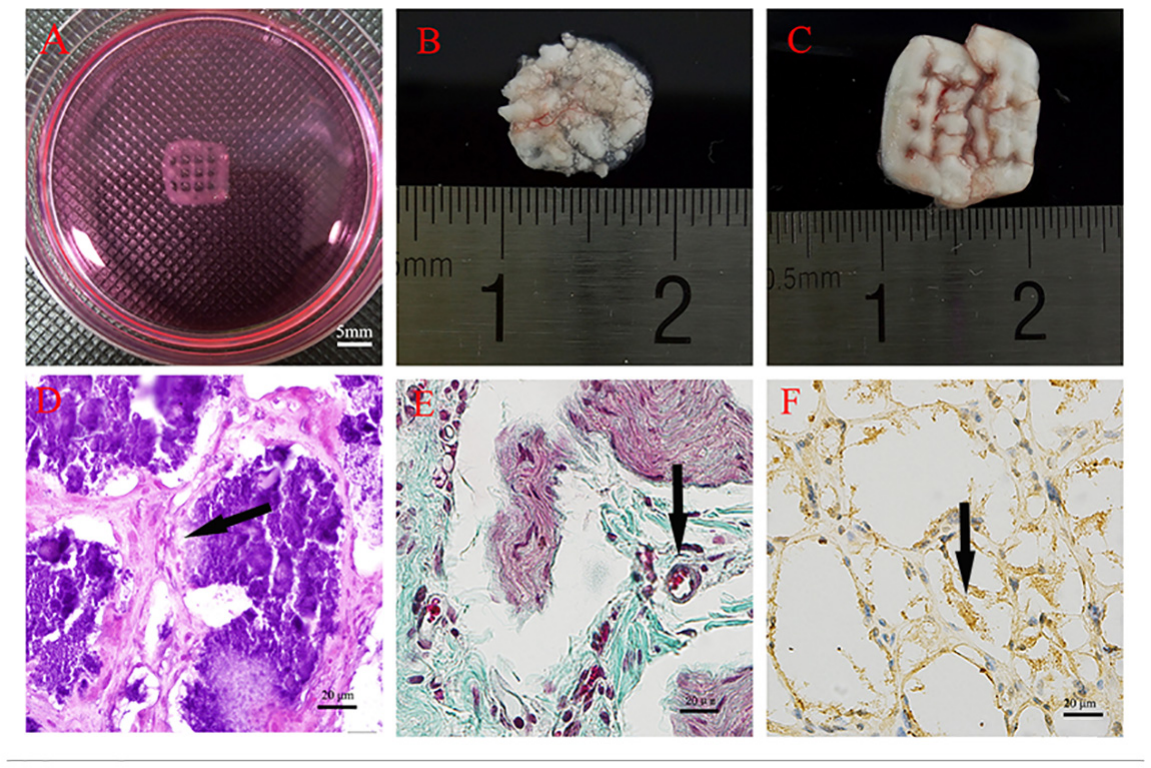
Ectopic bone formation. A: The 3D bioprinted construct before implantation. B: The degraded material without hASCs. C: The 3D bioprinted construct containing hASCs. D: H&E staining of paraffin-embedded sections at 8 weeks after implantation showing obvious new bone matrix formation. E: Masson trichrome staining showing new bone matrix formation. The arrow indicatesblood vessels. F: *OCN* immunohistochemical staining showing obvious *OCN* expression in the surrounding cells (see S4 and S5 Figs).

After 8 weeks of implantation, the printed 3D constructs (Fig 11A) without cells degraded, leaving a small amount of irregular materials (Fig 11B). Printed constructs containing cells remained in the original shape, and there were many blood vessels exhibiting ingrowth into the apertures of the constructs (Fig 11C). Paraffin-embedded sections were observed under a light microscope after H&E and Masson trichrome staining. The 3D printed construct containing hASCs exhibited obvious bone matrix formation, replacing part of the degraded material (Fig 11D and 11E). *OCN* immunohistochemical staining showed that *OCN* was distributed in the surrounding cells (Fig 11F).

## Discussion

This study focused on osteogenic differentiation of 3D bioprinted constructs consisting of hASCs. We investigated the osteogenic differentiation potential of hASCs after printing *in vitro* and *in vivo*.

Although many studies have reported the osteogenic differentiation of 3D biofabricated constructs, the seed cells used in all of these reports have been BMSCs [23–26]. Indeed, seed cells are one of the most important factors in 3D bioprinting technology [27]. BMSCs have obvious multipotent differentiation ability and are the most commonly used seed cells in bone tissue engineering. However, the bone marrow extraction process is complex and causes a great amount of distress for patients, despite the small amount of cells yielded from the extraction procedure. Thus, applicability of this technology is greatly limited.

hASCs, which were used as seed cells in this study, possess similar multipotent differentiation abilities as BMSCs, and there is an abundant source of adipose tissue from which large quantities of hASCs can be obtained. Based on these advantages, if the primary cells directly serve as seed cells, the applicability and safety of engineered bone tissue will be greatly enhanced [28]. The results of the current study showed that after a short duration of *in vitro* culture, 300 mL of adipose tissue could produce about 6 × 10^7^ primary hASCs possessing multipotent osteogenic and adipogenic differentiation potential. According to previous studies, this number of cells is sufficient for the construction of small-scale tissue-engineered complexes. Therefore, the application of hASCs as seed cells for tissue engineering is feasible, and further studies are needed to explore whether hASCs still possess the ability to form new bones in the 3D bioprinted constructs.

The materials used in 3D bioprinting technology are one of the most important factors affecting the tissue engineering process. Many types of biomaterial scaffolds, such as alginate, fibrin, and gelatin, can be fabricated [29]. The alginate used in our study is a type of naturally occurring anionic polymer that has many attractive characteristics for biomedical applications, including low toxicity, low cost, and excellent biocompatibility [30]. Due to the broad range of viscosities at room temperature, alginate-based hydrogels are attractive for 3D bioprinting. Alginate-based hydrogels exhibit appropriate viscosity and gelling speed as needed for accurate printing [31,32].

In this study, we used an alginate/gelatin mixture. Gelatin plays an important role in 3D bioprinted tissue constructs and can regulate the viscosity of the hydrogel to enable extrusion and shaping. Approximately 40% of gelatin is retained in the hydrogel immediately after cross-linking, and about 20% of that gelatin is then retained in the hydrogel over 7days of culture. This remaining gelatin can support cell spreading and adhesion [33]. We used different alginate/gelatin compositions during preliminary experiments. The results showed that increasing the proportion of alginate blocked the spread of the deposited hydrogel, whereas increasing the proportion of gelatin resulted in increased viscosity that was more conducive to 3D shaping [34]. Therefore, in order to ensure that the hydrogel could be smoothly printed onto the platform and maintain its shape until cross-linking via CaCl_2_, the optimal ratio of alginate to gelatin must be determined. Based on a previous study, we used hydrogel consisting of 2 wt% alginate and 8 wt% gelatin. SEM images showed that this hydrogel exhibited an uneven porous internal structure and average pore diameter of 28.47 μm. The experimental results indicated that this type of hydrogel complex met the requirements for 3D bioprinting and that cells in this the hydrogel still had proliferation and osteogenic differentiation capacity.

In our study, we used 3D bioprinting technology, which had the capacity to precisely place cells, biological factors, and biomaterial scaffolds into the desired 3D locations with computer control and is thus one of the most promising additive manufacturing (AM) approaches for tissue engineering [3,16,35,36]. The results of our study showed that hASCs were uniformly distributed within the alginate/gelatin 3D constructs. Thus, uniform distribution of cells was more conducive to the formation of uniform bone tissue. The cell viability results showed that cell survival rates in the 3D construct were 89% and 87% on days 1 and 7 after printing, which were higher than the survival rates reported in previous studies [32,37]. The number of viable cells on day 7 was greater than that on day 1, consistent with cell proliferation assays. These results showed that the cells could maintain viability and could proliferate in the 3D scaffold. Thus, the cells may establish tissue structures by secreting extracellular matrix, and the scaffold will gradually be degraded and absorbed, ultimately forming new bone tissue.

The results of our *in vitro* study showed that osteogenesis-related genes (*RUNX2*, *OCN*, and *OSX*) were upregulated in the 3D constructs when cultured in OM compared with that cultured in PM. Similar results were observed for *OCN* and *RUNX2* protein. Moreover, the *in vivo* results showed obvious new bone matrix formation, and we found that the osteogenic marker *OCN* was highly expressed in 3D constructs containing hASCs. All of these results demonstrated that hASCs cells located within the hydrogel still had multiple differentiation potential after printing, consistent with other studies [6,16]. These results indicated that 3D bioprinted constructs consisting of hASCs may be useful for applications in bone tissue engineering. Thus, our data further supported the study of tissue engineering using 3D bioprinting technologies.

There were some limitations to our study. The hydrogels used in this study did exhibit the disadvantage of low strength. Moreover, the newly printed microfilament layer on the tissue construct adhered to the previously printed microfilament layer, and as a result, the final height of the printed construct may be insufficient, and the microfilament may not reach the desired diameter. Therefore, future studies are required to develop materials that can meet the requirements of cell viability in 3D bio-printed tissue constructs while maintaining sufficient strength.

## Conclusions

This study successfully established 3D bioprinted tissue constructs with high cell viability and demonstrated the 3D spatial distribution of cells within this tissue construct. Additionally, we optimized the key parameters for hASCs-based 3D bioprinting. Our experiments confirmed that the 3D bioprinted constructs consisting of hASCs could promote mineralized matrix formation. After the bioprinting process, stem cells retained their capacity for proliferation and osteogenic differentiation. This study provided reliable evidence for the application of 3D bioprinting technology in bone tissue engineering. Our research group will continue subsequent research for development of clinically applicable hASCs-based 3D bioprinted tissue constructs to repair large-area bone defects.

## Supporting Information

S1 Fig SEM image showing the porous internal structure of the alginate/gelatin hydrogel. (TIF)

S2 Fig The bioprinted construct observed by an inverted light microscope (40×). (TIF)

S3 Fig Distribution of hASCs in the bioprinted tissue constructs (100×). (TIF)

S4 Fig The bioprinted 3D constructs without hASCs. (TIF)

S5 Fig The bioprinted3D constructs with hASCs. (TIF)
