# Supplementary figures and images for: Osteogenic Differentiation of Three-Dimensional Bioprinted Constructs Consisting of Human Adipose-Derived Stem Cells In Vitro and In Vivo
Source: PLoS One. 2016 Jun 22;11(6):e0157214. doi: 10.1371/journal.pone.0157214 (PMC4917247; doi:10.1371/journal.pone.0157214)

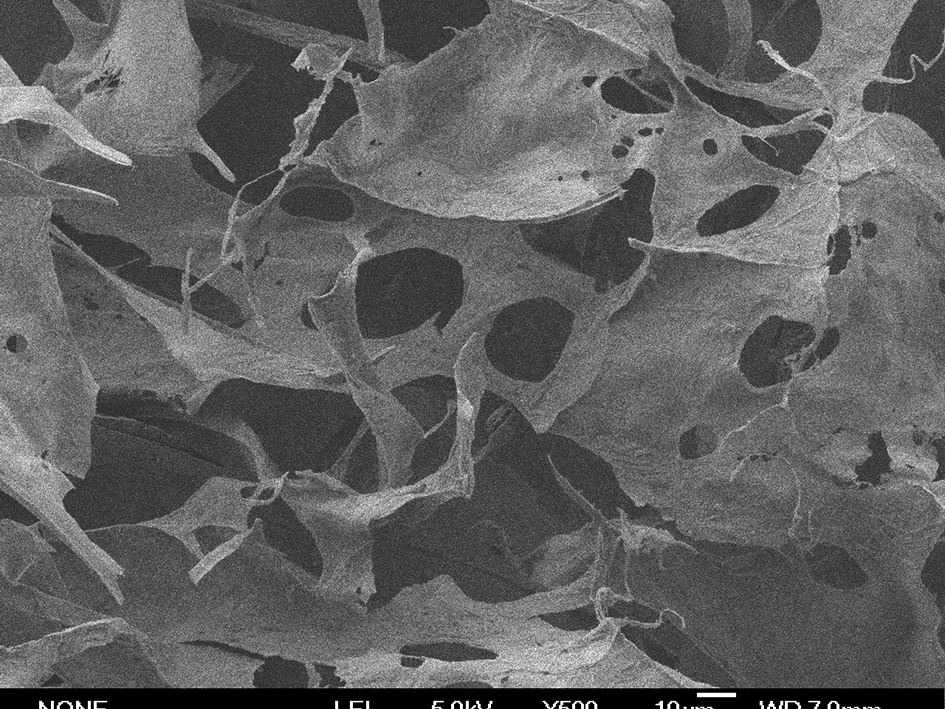

Supplement: S1 Fig — (TIF) [file pone.0157214.s001.tif]

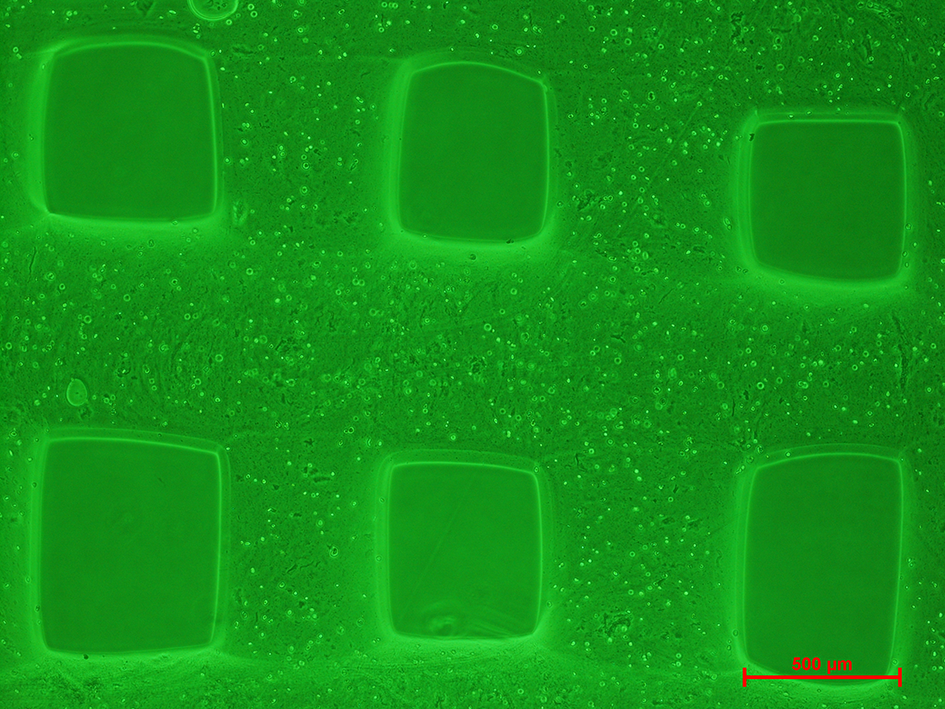

Supplement: S2 Fig — (TIF) [file pone.0157214.s002.tif]

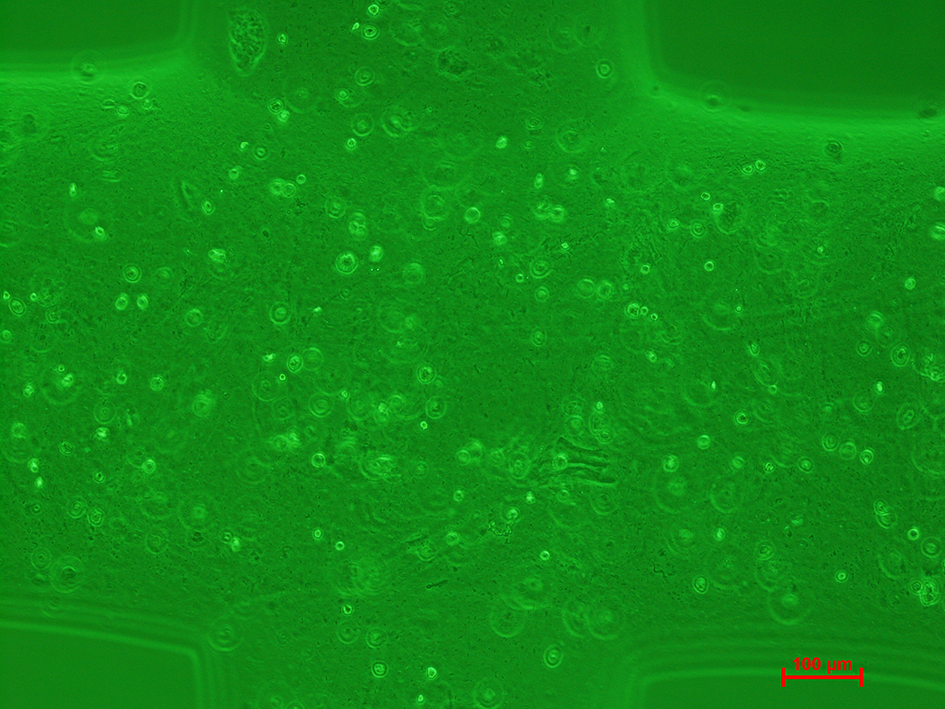

Supplement: S3 Fig — (TIF) [file pone.0157214.s003.tif]

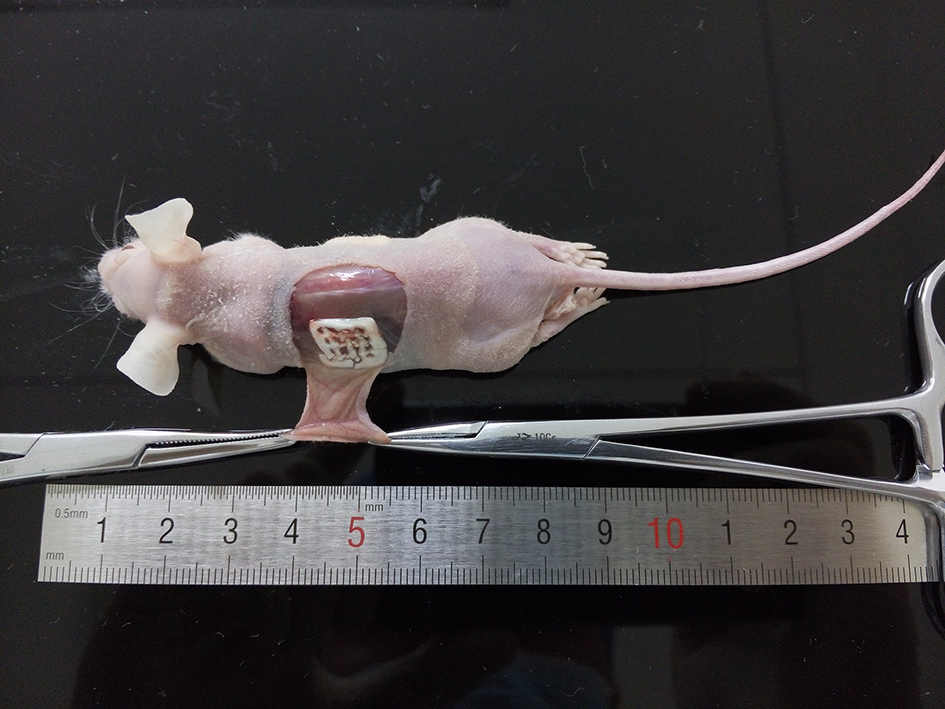

Supplement: S4 Fig — (TIF) [file pone.0157214.s004.tif]

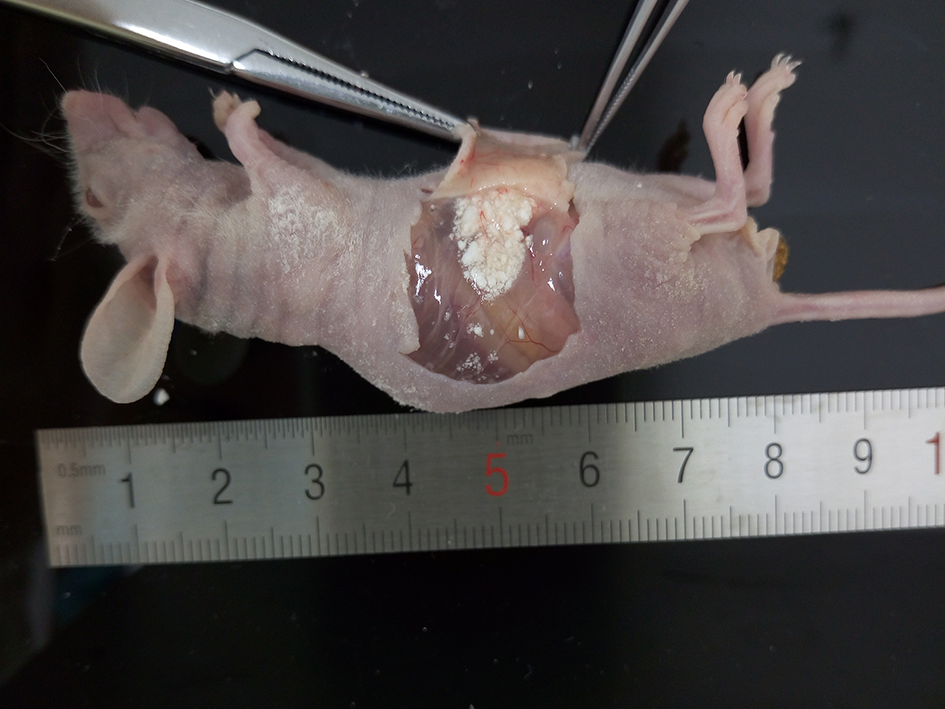

Supplement: S5 Fig — (TIF) [file pone.0157214.s005.tif]
